# Crosstalk between RNA m^6^A Modification and Non-coding RNA Contributes to Cancer Growth and Progression

**DOI:** 10.1016/j.omtn.2020.08.004

**Published:** 2020-08-08

**Authors:** Fengsheng Dai, Yongyan Wu, Yan Lu, Changming An, Xiwang Zheng, Li Dai, Yujia Guo, Linshi Zhang, Huizheng Li, Wei Xu, Wei Gao

**Affiliations:** 1 Shanxi Key Laboratory of Otorhinolaryngology Head and Neck Cancer, First Hospital of Shanxi Medical University, Taiyuan 030001, P. R. China; 2 Shanxi Province Clinical Medical Research Center for Precision Medicine of Head and Neck Cancer, First Hospital of Shanxi Medical University, Taiyuan 030001, P. R. China; 3 Department of Otolaryngology Head & Neck Surgery, First Hospital of Shanxi Medical University, Taiyuan 030001, P. R. China; 4 Key Laboratory of Cellular Physiology, Ministry of Education, Shanxi Medical University, Taiyuan 030001, P. R. China; 5 Department of Otolaryngology Head & Neck Surgery, The First Hospital of Jinzhou Medical University, Jinzhou 121001, P. R. China; 6 Department of Head and Neck Surgery, Cancer Hospital, National Cancer Center, Chinese Academy of Medical Sciences & Peking Union Medical College, Beijing 100021, P. R. China; 7 Department of Thyroid Surgery, Second Affiliated Hospital of Zhejiang University, Hangzhou 310009, P. R. China; 8 Department of Otolaryngology Head & Neck Surgery, Dalian Municipal Friendship Hospital of Dalian Medical University, Dalian 116100, P. R. China; 9 Department of Head & Neck Surgery, Shandong Provincial ENT Hospital Affiliated to Shandong University, Jinan 250022, P. R. China; 10 Shandong Provincial Institute of Otolaryngology, Jinan 250022, P. R. China; 11 Key Laboratory of Otolaryngology, Ministry of Health, Shandong University, Jinan 250022, P. R. China

**Keywords:** cancer, N6-methyladenosine, non-coding RNA, microRNA, long non-coding RNA, circular RNA

## Abstract

N6-methyladenosine (m^6^A) is the most common RNA modification and has an important role in normal development and tumorigenesis. The abnormal expression of m^6^A regulators can lead to an imbalance in m^6^A levels in cancer cells, leading to the dysregulated expression of oncogenes and tumor suppressor genes that may contribute to cancer development, patient response to chemoradiotherapy, and clinical prognosis. Recent studies demonstrate that non-coding RNAs are involved in epigenetic modification of both DNA and RNA in tumor cells, and may also affect the development and progression of cancer by targeting m^6^A regulators. In this review, we describe the functional crosstalk between m^6^A and non-coding RNAs, particularly microRNA, long non-coding RNA, and circular RNA, and illustrate their roles in tumor regulation. Finally, we discuss the significance of non-coding RNA and m^6^A modification in the diagnosis, treatment, and prognosis of cancer patients, as well as potential future research directions.

## Graphical Abstract

**Figure:**
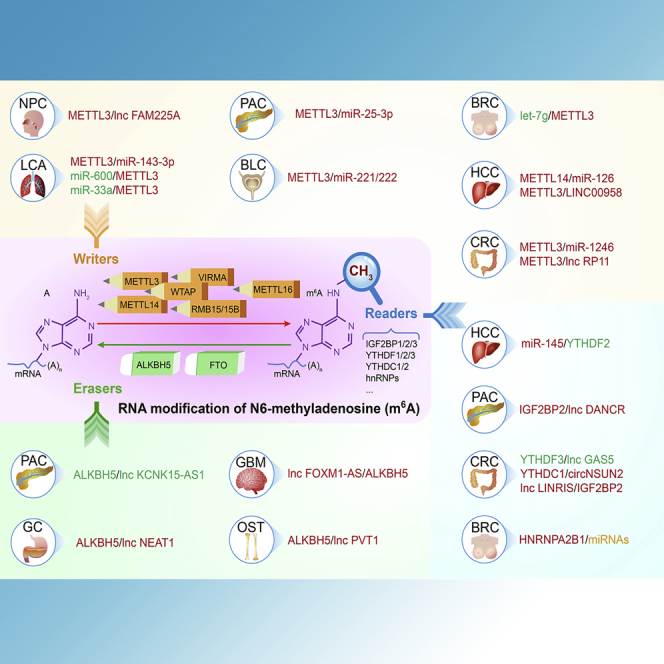

## Teaser

As a pivotal member of RNA modification, m^6^A plays an important role in the initiation and progression of cancer. However, the mechanism between m^6^A regulators and non-coding RNAs is largely unknown. Gao and colleagues summarize the crosstalk between m^6^A and non-coding RNAs in cancers and provide potential novel research directions.

## Main Text

According to recent global cancer statistics, cancer remains an important factor threatening human health.^1^ N6-methyladenosine (m^6^A) is the most common RNA modification and has attracted significant attention from researchers in the fields of tumorigenesis and development.^2^^,^^3^ Since the discovery of m^6^A in the early 1970s, studies have shown that it accumulates predominantly near the stop codons and 3′ untranslated regions (3′ UTRs) of mRNA.^4^, ^5^, ^6^, ^7^, ^8^ The abnormal expression of m^6^A regulators can lead to an imbalance in m^6^A levels in cancer cells, leading to the dysregulated expression of oncogenes and tumor suppressor genes that may contribute to cancer development, patient response to chemoradiotherapy, and clinical prognosis.^2^^,^^7^^,^^9^^,^^10^

Previous studies confirm that dysregulation of m^6^A regulators may be detected in precancerous lesions, highlighting their potential as molecular markers for the early diagnosis of cancer.^11^ Fat mass and obesity-associated protein (FTO) has been identified as an m^6^A demethylase that can selectively remove the m^6^A modification from target RNAs.^12^ A recent study showed that combination of FTO inhibitor and nilotinib can restrain the growth of leukemia and increase the sensitivity of leukemia cells to tyrosine kinase inhibitors, highlighting the potential therapeutic value of targeting m^6^A regulators in drug-resistant cancers.^13^ Although the FTO inhibitor, entacapone, has been approved by The Food and Drug Administration (FDA) for the treatment of cancer and other related diseases,^14^ specific inhibitors have not yet been identified for other m^6^A regulatory proteins.

**Figure 1:**
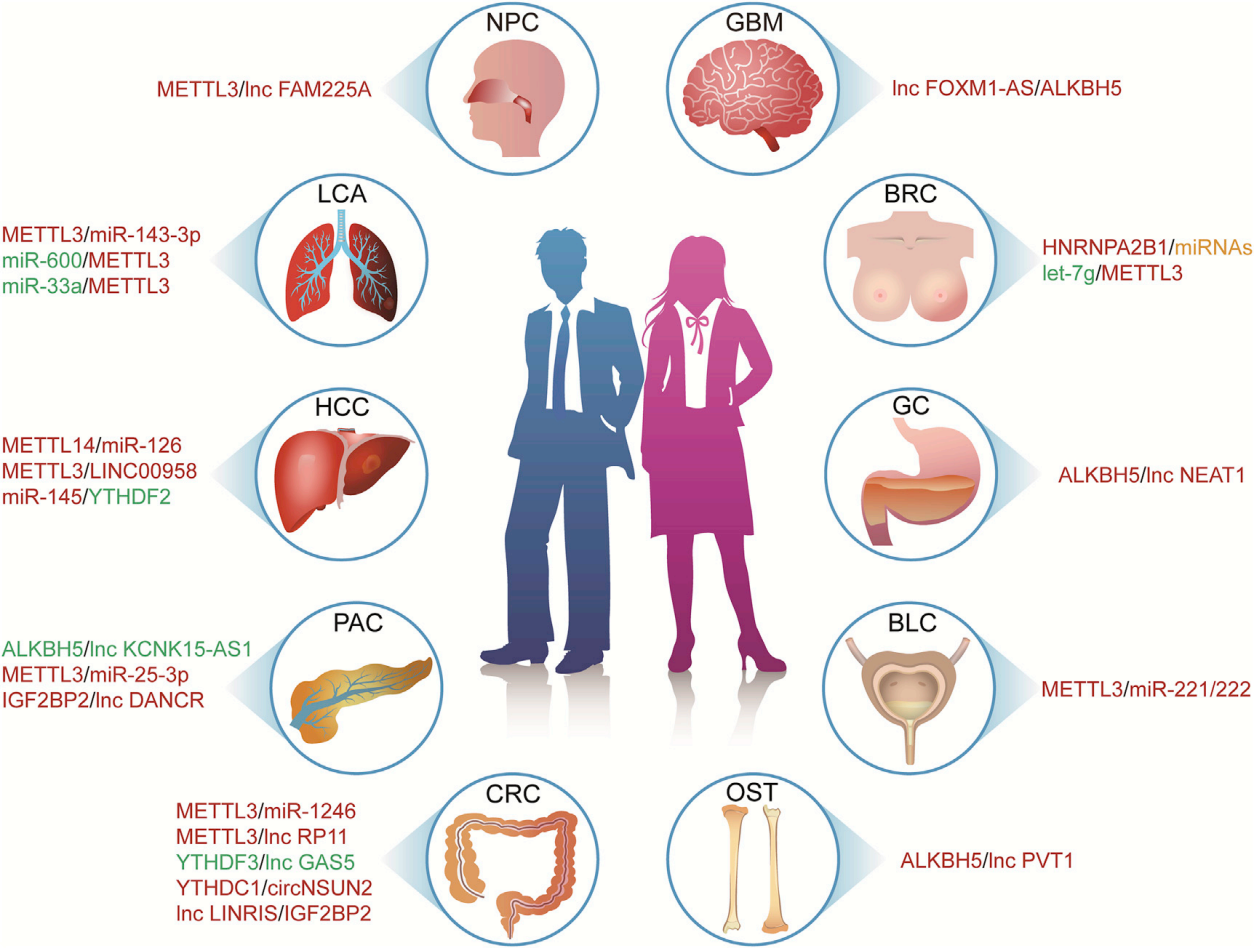
Non-coding RNAs and N6-methyladenosine (m^6^A) Modifications in Cancer Red font indicates an oncogenic role, green font indicates a tumor suppressor role, and yellow font indicates involvement of both oncogenic and tumor suppressor activities. BLC, bladder cancer; BRC, breast cancer; CRC, colorectal cancer; GBM, glioblastoma; GC, gastric cancer; HCC, hepatocellular carcinoma; LCA, lung cancer; NPC, nasopharyngeal carcinoma; OST, osteosarcoma; PAC, pancreatic cancer.

Although the function of m^6^A modification in cancer is becoming increasingly clear, its effect on protein translation and the molecular mechanisms underlying the effect of this mark on cancer progression remain unclear. Following the development of MeRIP-seq (methylated RNA immunoprecipitation sequencing) and miCLIP (m^6^A individual-nucleotide-resolution cross-linking and immunoprecipitation) technologies, researchers have found that non-coding RNAs, including long non-coding RNA (lncRNA), microRNA (miRNA), circular RNA (circRNA), transfer RNA, ribosomal RNA, and small nuclear RNA, are also capable of modifying DNA and RNA bases in cancer cells.^15^^,^^16^ Furthermore, non-coding RNA also participates in the regulation of m^6^A modification, thus affecting cancer progression (Figure 1).^3^^,^^17^

In this review, we describe the functional crosstalk between m^6^A and non-coding RNA, particularly miRNA, lncRNA, and circRNA, and illustrate how deregulation of these networks plays a role in tumors. Finally, we discuss the significance of non-coding RNA and m^6^A modifications in the diagnosis, treatment, and prognosis of cancer patients and possible future research directions.

### Overview of m^6^A Writers, Erasers, and Readers

**Figure 2:**
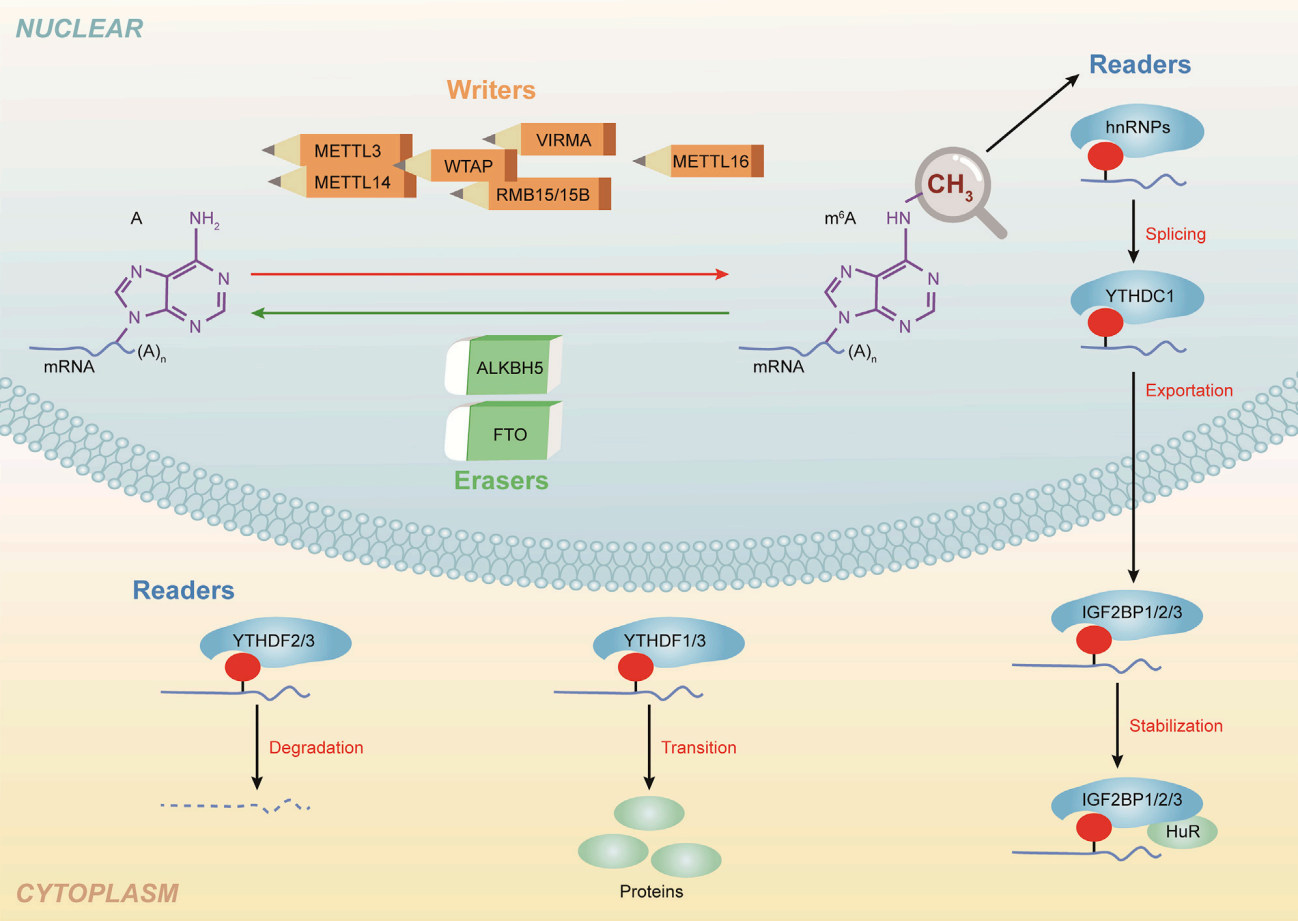
Summary of m^6^A Modification and Its Effects on mRNA Function m^6^A modification is a dynamic and reversible process. RNA can be methylated by “writers,” demethylated by “erasers,” and recognized by “readers.”

m^6^A modification is a dynamic and reversible process that has a critical role in regulating RNA stability, splicing, and translation. This modification is controlled by regulatory proteins referred to as “writers,” “erasers,” and “readers” (Figure 2). Epigenetic writers included methyltransferase-like 3/14/16 (METTL3/14/16), wt1-associated protein (WTAP), RNA binding motif protein 15/15B (RBM15/15B), and vir-like m^6^A methyltransferase-associated protein (VIRMA, also known as KIAA1429). METTL3/14 can form complexes to cause m^6^A methylation to be written into mRNA,^18^ and WTAP aids METTL3/14 to locate nuclear spots and maintain the catalytic activity of m^6^A methyltransferase *in vivo*.^19^ Meanwhile, METTL3 expression is essential for WTAP protein homeostasis.^20^ Moreover, RBM15, RBM15B, and VIRMA have roles in the regulation of METTL3 and METTL14 activity.^21^

Erasers include FTO and AlkB homolog 5 (ALKBH5). These proteins can selectively remove the m^6^A mark targeting mRNA through a series of complex intermediate reactions, thereby affecting tumor-specific biological processes.^22^ In 2001, researchers from the laboratory of Chuan He confirmed that FTO is an important DNA and RNA demethylase, particularly for m^6^A demethylation.^12^ The oncogenic role of FTO has since been confirmed in numerous cancers, including cervical cancer, breast cancer (BRC), and gastric cancer (GC).^23^, ^24^, ^25^

To date, several hypotheses have inferred that m^6^A modifications function by altering RNA structure or recruiting m^6^A readers. The most common readers include m^6^A RNA binding protein 1/2/3 (YTHDF1/2/3), YTH domain-containing 1/2 (YTHDC1/2), insulin-like growth factor 2 mRNA binding proteins 1/2/3 (IGF2BP1/2/3), heterogeneous nuclear ribonucleoproteins (HNRNPs), and zinc-finger CCCH domain-containing protein 13 (ZC3H13).^26^ Reader proteins function by binding to m^6^A sites on the target RNA and mediating its modification, thereby controlling RNA fate.^26^^,^^27^

In addition to revealing the functional roles and mechanisms of m^6^A RNA methylation in various cancers, recent studies highlighted the impact of m^6^A RNA methylation regulators on the diagnosis and prognosis of cancer patients (Table 1). Du et al.^10^ performed univariate Cox regression analysis to evaluate the clinical prognostic values of m^6^A RNA methylation regulators in glioblastoma (GBM), and revealed that HNRNPC, ALKBH5, and ZC3H13 are favorable prognostic markers, whereas FTO is an unfavorable prognostic marker for GBM. Furthermore, METTL3, YTHDC2, and YTHDF2 were identified as independent predictors of overall survival in liver cancer (LC).^28^ Moreover, Zhuang et al.^29^ built a 10-gene risk score model in lung adenocarcinoma (LUAD) through combined analysis of expression levels of m^6^A RNA regulators and clinicopathological characters. They found that the expression patterns of *ALKBH5*, *FTO*, *HNRNPC*, *YTHDF2*, *YTHDF1*, *YTHDC2*, *RBM15*, *KIAA1429*, *WTAP*, and *METTL3* were correlated with TNM stage, lymph node stage, and sex, as well as the living status of patients with LUAD. A two-gene signature consisting of *METTL3* and *METTL14* was identified as an independent prognostic indicator for distinguishing clear cell renal cell carcinoma (ccRCC) patients.^30^ These studies suggested that m^6^A regulators are potential diagnostic and prognostic markers for various cancers.

**Table 1:** Impact of m^6^A Modification Regulator on Diagnosis and Prognosis of Cancer Patients

Cancer Type	m^6^A Regulator	Diagnosis/Prognosis	References
GBM	HNRNPC, ZC3H13, ALKBH5	unfavorable prognostic marker	^10^
	FTO	favorable prognostic marker	
LC	METTL3, YTHDC2, YTHDF2	unfavorable prognostic marker	^28^
LUAD	ALKBH5, HNRNPC, YTHDF2, YTHDF1, YTHDC2, RBM15, KIAA1429, WTAP, METTL3, FTO	diagnostic marker, prognostic marker	^29^
ccRCC	METTL3	unfavorable prognostic marker	^30^
	METTL14	favorable prognostic marker	

### m^6^A Modification of Non-coding RNA

**Figure 3:**
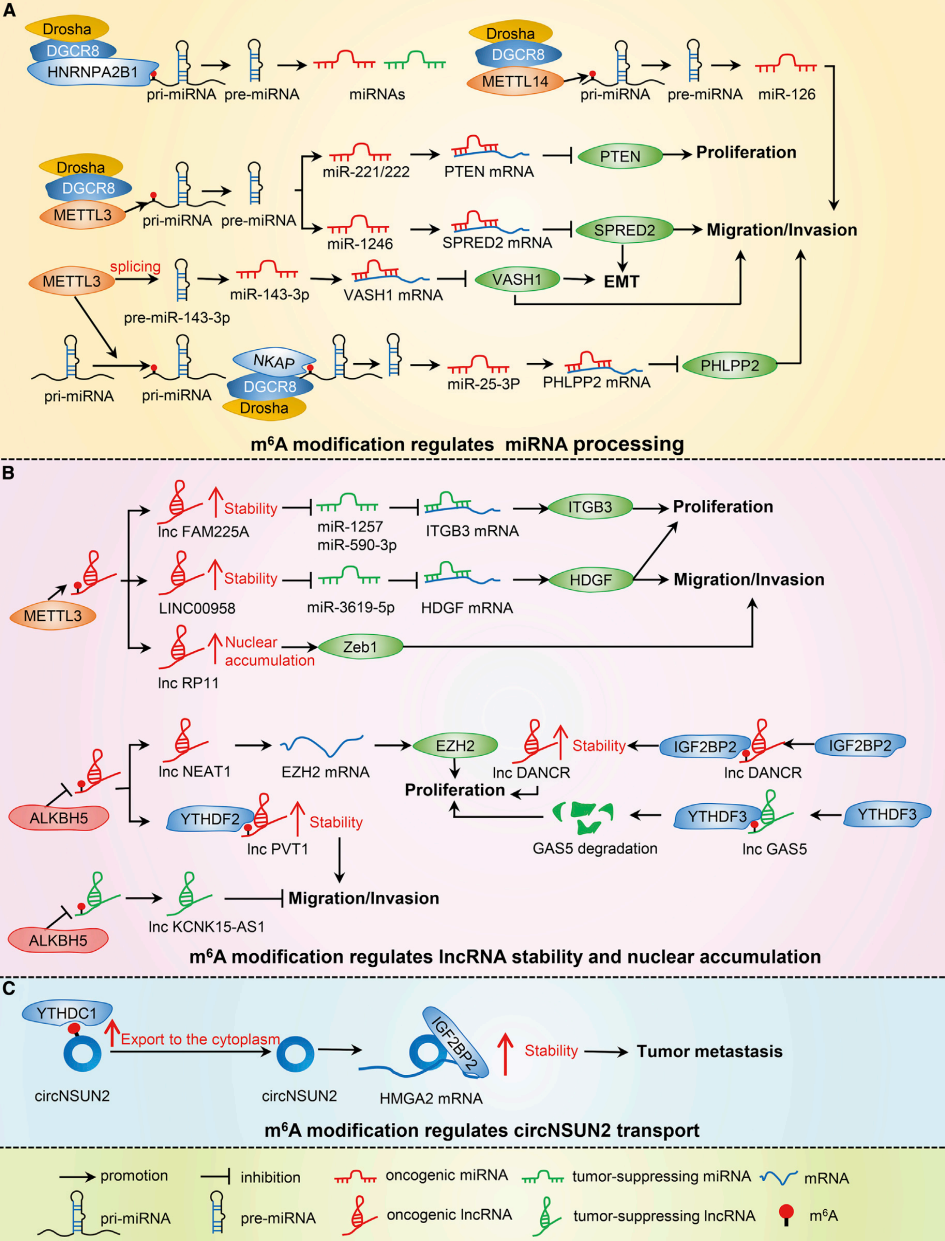
m^6^A Modifications in Non-coding RNA (A) m^6^A modification regulates miRNA processing. (B) m^6^A modification regulates lncRNA stability and nuclear accumulation. (C) m^6^A modification regulates circNSUN2 transport.

Non-coding RNAs comprise a large class of RNA transcripts without protein-coding potential that regulate gene expression and are important regulators of cancer cell proliferation, apoptosis, migration, immune response, and autophagy.^31^ m^6^A modification of non-coding RNA regulates important processes controlling RNA function, including processing, stability, and transport (Figure 3).^32^

### m^6^A Modification of miRNA

On the basis of our current understanding, miRNA biogenesis can be divided into three steps.^33^ In the nucleus, RNA polymerase II or III transcribes miRNA-related genes into primary miRNA (pri-miRNA). pri-miRNA is transformed into precursor miRNA (pre-miRNA) by the microprocessor complex, DGCR8-Drosha, and is subsequently transported out of the nucleus by the exportin-5-Ran-GTP complex. Finally, the microprocessor component, Dicer, cleaves the pre-miRNA into mature mRNA in the cytoplasm.^34^^,^^35^ miRNAs have important roles in the regulation of gene expression, mainly through their association with AGO2 as part of the RNA-induced silencing complex (RISC), via binding to mRNA 3′ UTR, leading to degradation and inhibition of translation.^36^^,^^37^ In 2002, the Croce team first identified the role of miRNAs in cancer, demonstrating low expression of *miR-15* and *miR-16* in chronic lymphocytic leukemia patients.^38^ Since then, numerous studies have indicated that abnormal expression of miRNA underlies many pathological processes related to tumorigenesis.^39^ Notably, there is a strong association between m^6^A and miRNA binding sites in mammals.^4^

The synthesis and function of miRNAs may be affected by m^6^A modification at multiple levels (Table 2). Studies by Alarcón et al.^40^ indicate that heterogeneous nuclear ribonucleoproteins A2/B1 (HNRNPA2B1) can read m^6^A marks and enhance DGCR8 binding to pri-miRNA transcripts, affecting miRNA processing. Similarly, the m^6^A writer METTL14 can interact with DGCR8 and promote *miR-126* processing in an m^6^A-dependent manner in hepatocellular carcinoma (HCC).^41^ In bladder cancer, METTL3 is overexpressed and regulates the processing of *miR-221*/*miR-222* in an m^6^A-dependent manner via recruitment of DCGR8.^42^ METTL3 also promotes *pri-miR-1246* maturation via a similar mechanism and positively modulates tumor cell metastasis.^43^ Studies by Zhang et al.^44^ emphasize the importance of the m^6^A writer METTL3 on *miR-25-3p* maturation and identified NKAP as an m^6^A reader for *pri-miR-25* processing in pancreatic cancer (PAC). METTL3 can accelerate the brain metastasis of cancer cells and promote the splicing of *pre-miR-143-3p* to produce mature miRNA, and may be associated with Dicer in lung cancer (LCA).^45^ As previously discussed, m^6^A promotes miRNA maturation by regulating its processing, thereby enhancing the degradation and translational inhibition of downstream target mRNAs.

**Table 2:** m^6^A Modification Regulates miRNA Processing

m^6^A Regulator	Associated Cancer	Function	References
**Reader**			

HNRNPA2B1	BRC	promotes miRNAs processing	^40^

**Writer**			

METTL14	HCC	promotes the processing of miR-126 via DGCR8	^41^
METTL3	BLC	promotes the processing of miR-221/222 via DGCR8	^42^
METTL3	CRC	promotes the processing of miR-1246 by DGCR8	^43^
METTL3	PAC	promotes miR-25-3p maturation	^44^
METTL3	LCA	promotes splicing of pre-miR-143-3p	^45^

Interestingly, m^6^A modifications can also protect mRNA degradation mediated by miRNA. Müller et al.^46^ demonstrated that IGF2BP1 affects miRNA-directed decay of *SRF* mRNA, increasing SRF expression in an m^6^A-dependent manner. In colorectal cancer (CRC), IGF2BP2 maintains *RAF1* mRNA stability by blocking miRNA-mediated degradation, thereby increasing cancer cell proliferation.^47^ Furthermore, m^6^A modification of *AGO2* mRNA has also been reported to affect miRNA levels.^48^ In conclusion, m^6^A modification plays an important role in miRNA biogenesis, and the effects of m^6^A-mediated miRNA level variation require further investigation.

### m^6^A Modification of lncRNA

The role of lncRNA in tumor development is complex and diverse.^49^ lncRNAs may regulate gene transcription via binding to gene promoters,^50^ affect the variable splicing of RNA and maintain the normal function of intracellular organelles,^51^ act as miRNA sponges, relieving inhibition of miRNA target genes,^52^ and affect the stability and translation of mRNA via RNA interactions.^53^^,^^54^ They can also affect protein function by acting as a scaffold for protein-protein interactions, modulating their localization to chromatin, and regulating protein post-translational modifications and stability.^55^ lncRNAs are located in different subcellular structures, including the cell membrane, cytoplasm, nucleus, and paraspeckles, and their functions and regulatory mechanisms are closely related to their localization in cancer cells.^56^

m^6^A modification of lncRNA regulates numerous processes affecting cancer cell activity (Table 3). In HCC, METTL3-mediated m^6^A leads to upregulation of LINC00958 by enhancing its stability, thereby promoting cancer progression.^57^ METTL3 has also been shown to increase the stability of *FAM225A*, a lncRNA overexpressed in nasopharyngeal carcinoma (NPC), to promote tumorigenesis.^58^ Moreover, studies have shown that METTL3 can upregulate expression of *RP11* lncRNA by increasing its nuclear accumulation in CRC.^59^ The m^6^A eraser ALKBH5 has been shown to act as both a tumor suppressor and promoter, and has the ability to demethylate m^6^A on single-stranded RNA and DNA.^60^^,^^61^^,^^65^ As a tumor suppressor, ALKBH5 is significantly downregulated in PAC, and its expression is related to patient survival, as well as being an independent marker of prognosis. ALKBH5 can also regulate the expression of KCNK15-AS1 via demethylation, leading to inhibition of PAC cell migration and invasion.^60^ ALKBH5 also plays a role in tumor progression, promoting osteosarcoma (OST) cell proliferation via upregulation of lncRNA *PVT1*.^61^ Additionally, ALKBH5 is upregulated in GC cells and increases invasion and metastasis via inhibition of *NEAT1* methylation.^62^ The m^6^A readers IGF2BP2 and YTHDF3 are also involved in the regulation of lncRNA. IGF2BP2 is highly expressed in PAC and interacts with lncRNA *DANCR*, leading to an increase in its stability and promoting cancer cell proliferation.^63^ In a similar manner, YTHDF3 can negatively regulate *GAS5* lncRNA and promote progression of CRC.^64^

**Table 3:** m^6^A Modification Regulates lncRNA

m^6^A Regulator	Associated Cancer	Function	References
**Writer**			

METTL3	HCC	enhances LINC00958 stability	^57^
METTL3	NPC	increases lnc FAM225A stability	^58^
METTL3	CRC	increases lnc RP11 nuclear accumulation	^59^

**Eraser**			

ALKBH5	PAC	inhibits lnc KCNK15-AS1 methylation	^60^
ALKBH5	OST	decreases the m^6^A modification of lnc PVT1	^61^
ALKBH5	GC	decreases methylation of the lnc NEAT1	^62^

**Reader**			

IGF2BP2	PAC	increases lnc DANCR stability	^63^
YTHDF3	CRC	promotes decay of lnc GAS5	^64^

In the nucleus, lncRNAs may recruit regulatory proteins and interact with mRNAs or act as competing endogenous RNAs (ceRNAs), regulating the translation and stability of mRNA.^66^ Therefore, we infer that m^6^A modifications may affect similar regulatory functions of cytoplasmic lncRNAs. However, our understanding of m^6^A modifications of lncRNA is still limited.

### m^6^A Modification of circRNA

circRNA was first identified in eukaryotes nearly 40 years ago and was subsequently discovered in humans infected with hepatitis delta virus (HDV).^67^^,^^68^ Studies demonstrated that circRNA can specifically adsorb and bind miRNA, releasing the inhibition of miRNA on downstream target genes and directly binding proteins to modulate their function.^69^^,^^70^ In human cancer, circRNA regulates critical cellular processes, including proliferation, metastasis, differentiation, autophagy, and drug resistance.^71^, ^72^, ^73^, ^74^

Recent studies revealed that circRNA has the potential to be translated. Yang et al.^75^ demonstrated that m^6^A levels in circRNA can promote efficient initiation of protein translation from human cells. Other studies have shown that YTHDF1 and YTHDF2 can interact with circRNAs, and that METTL3 also affects circRNA m^6^A levels, suggesting that m^6^A is modified by the same machinery in both circRNAs and mRNAs.^76^ However, enrichment of m^6^A in circRNAs is mainly at the translation start site of their corresponding mRNAs, differing from mRNA.^76^ The m^6^A reader YTHDC1 has been shown to increase *circNSUN2* export to the cytoplasm (Table 4), leading to the formation of a *circNSUN2*-IGF2BP2-HMGA2 RNA-protein ternary complex that can stabilize *HMGA2* mRNA and enhance colorectal liver metastasis.^77^ Another study also reports that METTL3 can impact *circZNF609* m^6^A modification, and YTHDC1 regulates back-splicing of *circZNF609*, which highlights the critical role of m^6^A modification in *circZNF609* biogenesis and translation in HeLa cells.^78^ These studies provide a new perspective on m^6^A modification of circRNA.

**Table 4:** m^6^A Modification Regulates circRNA

m^6^A Regulator	Associated Cancer	Function	Reference
YTHDC1	CRC	increases circNSUN2 export to the cytoplasm	^77^

### Regulation of m^6^A Modification by Non-coding RNAs

#### miRNA Affects m^6^A Modification

**Figure 4:**
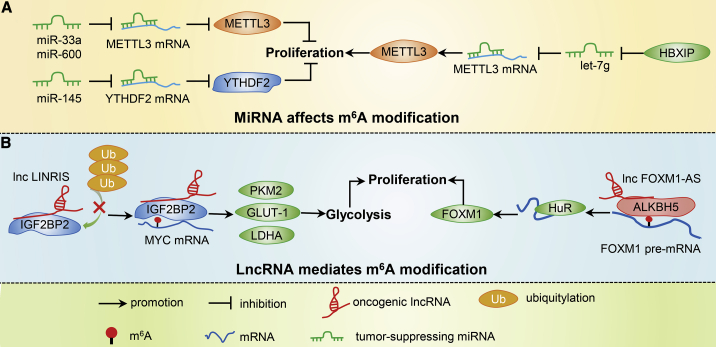
Overview of Non-coding RNA-Mediated Regulation of m^6^A Modifications in Cancer (A) miRNA affects m^6^A modification. (B) lncRNA mediates m^6^A modification.

Studies have shown that m^6^A modifications can be controlled by miRNA levels (Table 5). In LCA, *miR-600* decreases the expression of METTL3 and reverses the effect of METTL3 on cancer cell progression.^79^ In keeping with these findings, *miR-33a* targeting of the METTL3 3′ UTR leads to the downregulation of METTL3 expression and suppression of non-small cell lung cancer (NSCLC) proliferation.^80^ *Let-7g* miRNA can also inhibit METTL3 expression by targeting its 3′ UTR; moreover, HBXIP increases METTL3 expression by restraining *let-7g* in BRC.^81^ Yang et al.^82^ reveal that overexpression of *miR-145* strongly increases m^6^A levels via targeting of the 3′ UTR of YTHDF2 in HCC (Figure 4A). Together, these studies indicate that miRNAs may affect m^6^A modification by controlling the levels of m^6^A regulators.

**Table 5:** miRNA Affects m^6^A Modification

miRNA	Associated Cancer	Function	m^6^A Regulator	References
miR-600	LCA	downregulates the expression of METTL3	writer	^79^
miR-33a	NSCLC	decreases METTL3 through targeting its 3′ UTR	writer	^80^
let-7g	BRC	restrains METTL3 expression by targeting its 3′ UTR	writer	^81^
miR-145	HCC	inhibits YTHDF2 by targeting its 3′ UTR	reader	^82^

#### lncRNAs Regulate m^6^A Modification

As discussed above, m^6^A modification participates in lncRNA biogenesis and can affect functional activity. Conversely, lncRNAs can also affect m^6^A regulators and influence their function in cancer cells (Table 6). For example, the lncRNA *LINRIS* is upregulated in CRC cells and maintains IGF2BP2 protein stability via blocking the ubiquitination-proteasome pathway.^83^ ALKBH5 is highly expressed in primary GBM cell lines and promotes cancer cell proliferation. The lncRNA *FOXM1-AS* promotes the interaction between ALKBH5 and FOXM1, leading to demethylation of *FOXM1* mRNA and overexpression of FOXM1 (Figure 4B).^84^ These studies suggest that regulation of m^6^A modifications by antisense lncRNAs may be a common mechanism. As a regulatory subunit of IGF2BP1, the peptide RBRP encoded by *LINC0266-1* can recognize m^6^A modification via binding to IGF2BP1 and recruit stable RNA molecules to maintain the stability of *MYC* mRNA, thus promoting the occurrence and development of tumors.^85^ This study enriches our understanding of the effect of lncRNA on m^6^A modification.

**Table 6:** lncRNA Regulates m^6^A Modification

lncRNA	Associated Cancer	Function	m^6^A Regulator	References
LINRIS	CRC	stabilizes IGF2BP2	reader	^83^
FOXM1-AS	GBM	allows the interaction between FOXM1 and ALKBH5	eraser	^84^

### Conclusions

m^6^A regulators can modulate non-coding RNAs via multiple mechanisms, including regulation of pri-miRNA processing, affecting m^6^A-dependent ceRNA networks, promoting the nucleation of circRNA, and even by regulating the interaction between lncRNAs and proteins. These studies typically use poly(A)^+^ RNA for m^6^A mapping, excluding many regulatory ncRNA species that do not contain poly(A) tails. In addition, miRNA and lncRNA can also regulate m^6^A levels in cancer cells. miRNAs can target the corresponding mRNA of m^6^A regulators to silence their expression, thus altering m^6^A levels in cancer cells. In the nucleus, lncRNAs may act as scaffolds, providing a platform for other effector molecules to interact with m^6^A. Additionally, lncRNAs may be involved in maintaining the stability of m^6^A-related proteins. Notably, Huang et al.^86^ reported that *circSTAG1* can bind ALKBH5 and inhibit its nucleation, thus changing the total RNA m^6^A modification and increasing the m^6^A modification level of RNAs, including *FAAH* mRNA in the chronic unpredictable stress mouse hippocampus. This study provides the foundation for analyzing the relationship between circRNA and m^6^A modification. circRNA can not only be modified by m^6^A, but can also regulate the process of m^6^A modification by binding to m^6^A-modified proteins. However, the regulation of m^6^A modification by circRNA has not yet been reported in human cancer.

The crosstalk between non-coding RNAs and m^6^A modifications provides a new perspective for us to study normal development and tumorigenesis and to understand its complex regulatory network. Dynamic m^6^A modification of non-coding RNA represents a novel mechanism to regulate genetic information in cancer cells and adds to our understanding of how m^6^A modifications regulate RNA and downstream biological processes. The finding that m^6^A levels can in turn be regulated by non-coding RNAs enriches our understanding of non-coding RNA molecular networks in cancer progression. Together, these findings provide new directions to study the mechanism of non-coding RNA in tumorigenesis and development.

### Future Prospects

According to the central dogma, generation of the entire proteome from the genome requires regulation at four main stages: RNA production (including epigenetic regulation and transcriptional regulation), RNA degradation, protein production (translation regulation), and protein degradation. Of these, regulation of translation constitutes the most important mode of regulation in the cell, accounting for more than half of all regulatory events.^87^ To date, there have been few studies in this field because of the limitations of the current research methods and other factors.

At present, RNA methylation and translation-omics are new directions in epigenetic research and will provide important insights into the novel mechanisms governing normal physiological and abnormal cellular processes. Research on the functional interaction of non-coding RNA and m^6^A modification deserves particular attention. It is worth noting that studies investigating the crosstalk between non-coding RNA and m^6^A regulators typically involve members transcribed from different parental genes. Whether non-coding RNA and m^6^A regulators transcribed from the same gene can interact to regulate downstream target genes through positive or negative feedback loops will be of interest in the future. Studying the cross regulation of non-coding RNA and m^6^A modification will facilitate the discovery of critical targets for the diagnosis and treatment of cancer patients, which is the ultimate goal of personalized medicine. Given the involvement of these regulatory processes in normal development and other diseases, these findings are likely to have widespread applications, although the specific mechanisms in these cell types require further exploration.^48^^,^^88^

## Author Contributions

W.G., W.X., and Y.W. conceived this manuscript. F.D., Y.L., C.A., and L.Z. collected and prepared the related references. F.D., Y.W., and Y.L. drafted the manuscript. Y.G. and F.D. drew the figures. Y.L., L.D., and L.Z. performed data analysis and tabulation. W.G., Y.W., H.L., and W.X. supervised and revised the manuscript. All authors read and approved the final manuscript.

## Conflicts of Interest

The authors declare no competing interests.
